# FDA’s proposed rule and its regulatory impact on emerging and reemerging neglected tropical diseases in the United States

**DOI:** 10.1371/journal.pntd.0012116

**Published:** 2024-05-09

**Authors:** Alberto E. Paniz-Mondolfi, Juan David Ramírez

**Affiliations:** Molecular Microbiology Laboratory, Department of Pathology, Molecular and Cell-Based Medicine, Icahn School of Medicine at Mount Sinai, New York, New York, United States of America; University of Geneva Hospitals, SWITZERLAND

## Abstract

Diagnosing infectious diseases significantly influences patient care, aiding in outbreak identification, response, and public health monitoring. However, the range of FDA-approved molecular tests remains notably limited, especially concerning neglected tropical diseases (NTDs). Drawing upon our experience as one of the largest healthcare networks in the greater New York metropolitan area, this viewpoint manuscript aims to spotlight the existing diagnostic landscape and unmet clinical needs for 4 emerging NTDs increasingly prevalent in the United States, additionally, it delves into the possible adverse effects of the FDA’s Proposed Rule on Laboratory-Developed Tests for these clinical conditions and the broader spectrum of NTDs.

In the post–Coronavirus Disease 2019 (COVID-19) era, infectious diseases have become a major focus for the development of molecular diagnostic tests, evolving into a dynamically growing field that has begun to revolutionize clinical diagnosis, particularly in urgent clinical scenarios requiring a rapid diagnostic method with high sensitivity and specificity, ideally accessible to the wider population [1,2]. This becomes even more crucial in the context of emerging and neglected infectious diseases, now recognized as endemic in the United States of America [3], where diagnostic options are either nonexistent or limited to select reference centers. Such is the case for viral infections like Mpox [4] and dengue [5], and parasitic infections such as American Trypanosomiasis (Chagas disease) [6], leishmaniasis [7], and malaria [8].

Clinical laboratories play a crucial role in the healthcare system, particularly in managing the dynamic and intricate challenges posed by infectious diseases [9]. Many of the tests available in the field of infectious diseases have evolved from research use-only (RUO) tests to in vitro diagnostic (IVD) products, ultimately gaining approval from the Food and Drug Administration (FDA) and marked as FDA-cleared [10]. In the USA, IVDs are regulated by the FDA following a device categorization system ranging from I to III, with those categorized as I considered low-risk and those as II and III considered high-risk, thus requiring premarket approval [11,12].

Conversely, at the federal level, laboratory-developed tests (LDTs) historically have not been subject to FDA regulation but instead fall under the jurisdiction of the Centers for Medicare and Medicaid Services (CMS) through the Clinical Laboratory Improvement Amendments (CLIA) [12,13]. In this sense, CLIA constitutes the primary regulatory framework for LDTs, a subject over which the FDA is now seeking jurisdiction.

On September 29, 2023, the FDA unveiled a proposed rule with the objective of bolstering the safety and efficacy of diagnostic tests. This rule aims to explicitly define IVD devices, including those developed by academic clinical laboratories, as devices regulated under the Federal Food, Drug, and Cosmetic Act. Furthermore, the FDA is proposing a new policy to increase supervision of LDTs, indicating a shift away from its previous approach of exercising discretion in enforcing regulations for most LDTs.

These claims regarding the jurisdiction, oversight, and surveillance of LDTs by the FDA are not new and date back to as far as 2007 with the introduction of a draft guidance for In Vitro Diagnostic Multivariate Index Assays (IVDMIAs) [14]. This was followed by 2 draft guidance documents proposing an initial regulatory framework for LDTs in 2014 [15,16], further followed by the bipartisan proposal of the Diagnostic Accuracy and Innovation Act (DAIA) in 2017 [17]. Subsequent to this, the introduction of the Verifying Accurate Leading-Edge IVCT Development (VALID) Act in 2018 [18] and its most recent reintroduction before Congress in 2021 continue to fuel discussions in this realm. Details concerning these timelines and specifics, from a legislative and regulatory standpoint regarding LDTs, fall outside the scope of this article but have been extensively and meticulously reviewed elsewhere [12,19–21].

While the FDA’s viewpoint in its mission to minimize potential risks associated with LDT-based diagnosis is commendable and understandable, the proposed FDA rule constitutes a setback that will negatively impact the diagnostic field for infectious diseases, particularly neglected diseases currently on the rise in the US. Based on our personal experience of collaborating with the FDA in the validation and subsequent Emergency Use Authorization (EUA) approval of our saliva-based diagnostic SARS-CoV-2 PCR assay [22], we can attest to the immense scientific rigor and professional competence demonstrated by the FDA in their evaluation processes.

However, we also acknowledge that other regulatory bodies exhibit an equivalent level of demand and rigor. In our case, we specifically refer to the New York State Department of Health (NYSDOH) as a regulatory body that, from our personal viewpoint, demonstrates not only a level of rigor comparable to the FDA but also an equivalent standard of assessment in validation and the monitoring of performance through stringent quality control programs and regular proficiency testing [23].

In this regard, the proposed rule on LDTs seems redundant within the regulatory framework currently governing clinical laboratories under federal administration and supervision, such as CLIA, along with other professional organizations like College of American Pathologists (CAP), The Joint Commission (TJC), and various state health departments [19].

The COVID-19 pandemic posed many challenges for FDA, despite its well-established programs for reviewing IVD products, quickly overwhelming the FDA’s resources and decision-making capacity [19,24]. In this context, a valid question arises: Can the FDA manage an overwhelming number of premarket application submissions nationwide, similar to the scenario faced during the peak of the pandemic with the overflow of EUA submissions? Such a situation would hinder many laboratories from timely enabling their tests, thereby limiting patients’ access to diagnostic tests, particularly in the context of outbreaks caused by emerging infectious agents, as seen in the recent Mpox multicountry outbreak. In this scenario, the rapid implementation by the US Laboratory Response Network [25] and other laboratories, mainly in academic centers (such as ours through an LDT) (see S1 Table) [26], played a crucial role in providing expanded test access and effectively responding to the outbreak through surveillance [27] and response measures.

Aligned with the points mentioned earlier, the regulatory hurdles introduced by the proposed ruling could not only restrict access to timely diagnosis but also confine diagnostic operations to a handful of reference laboratories, thereby prolonging turnaround times. This limitation would impede the essential agility needed to address emerging pathogens, which are characteristic of the constantly shifting and swiftly evolving landscapes that fuel epidemics and disease outbreaks.

Even more concerning is the outlook for addressing diagnostic scenarios involving silent emerging pathogens. This is particularly relevant for diseases like Chagas, leishmaniasis, and blood parasites (particularly babesiosis and malaria) where the current diagnostic arsenal in the USA is extremely limited. In addition, the advent of cutting-edge technologies, like metagenomics, is contributing to swift pathogen discovery [28]. These innovations play a crucial role in identifying emerging threats that pose a risk to the US population. Here, we aim to share our specific experiences within the Mount Sinai Health System in New York City, illustrating how the FDA rule can influence the development of LDTs for neglected tropical diseases (NTDs).

## Chagas disease

For instance, Chagas disease is expected to endure continued underdiagnosis and an underestimation of its prevalence in the US [6]. Presently, most diagnosed Chagas disease cases are presumed to have originated outside the US or through congenital transmission, organ transplantation, or blood transfusion. However, substantial evidence suggests the potential for vectorial transmission and endemicity of Chagas disease within the country [6]. Notably, as of 2014, 11 species of triatomine bugs have been documented in 27 mainland US states and Hawaii, some of which exhibit a preference for human habitats. In regions like Texas, at least half of these bugs have been reported to carry *Trypanosoma cruzi*, the causative agent of Chagas disease. This situation is compounded by the presence of various mammalian wildlife and synanthropic species found to harbor *T*. *cruzi*, acting as reservoirs for the parasite. The coexistence of competent disease vectors and numerous mammalian reservoirs significantly contributes to the risk of human transmission and infection within the US [6].

It is important to note that at Mount Sinai Molecular Microbiology Laboratory, we have undertaken efforts to address these challenges. Specifically, we have developed and validated a LDT for pan-stage real-time PCR, enabling the quantitation of *T*. *cruzi* parasitic loads in blood samples (see S1 Table) [29].

Our endeavors in this area have resulted in practical implications from a laboratory perspective, particularly regarding the utilization of all known *T*. *cruzi* stages to construct standard curves for the assessment of *T*. *cruzi* pan-stage levels [29]. This innovation signifies a significant step toward enhancing diagnostic capabilities for Chagas disease especially in cases involving the infection reactivation in post-donor–recipient scenarios and underscores the critical importance of accessibility to such diagnostic tools in the face of potential regulatory barriers posed by the FDA’s proposed ruling. Additionally, beyond clinical diagnosis, the implementation of this pan-stage PCR can also provide a necessary alternative for entomological and veterinary surveillance, thereby enhancing the evaluation of *T*. *cruzi* prevalence in triatomine and animal populations and aiding in assessing several crucial facets of transmission dynamics.

Considering the necessity for expanded Chagas disease screening across the nation, the anticipated increase in testing underscores the critical need for suitable test options. The FDA’s proposed ruling may further limit access to these crucial diagnostics, exacerbating the challenge of diagnosing diseases like Chagas and impeding efforts to combat their spread within the US.

## Leishmaniasis

Cutaneous leishmaniasis, though endemic in certain southwest states, is of particular concern [7]. Documented autochthonous transmission in Texas, the emergence of a US-endemic *Leishmania* species [30] and the presence of competent vectors, including *Lutzomyia shannoni*, *Lu*. *anthropophora*, and other species like *Lu*. *diabolica*, emphasize the potential for further endemism extension across the country [7]. This scenario stresses the need for enhanced diagnostic tools to effectively address and mitigate the spread of this disease.

Additionally, visceral leishmaniasis, although not considered endemic, poses a significant risk of establishment within the US. The presence of competent sandfly vectors, combined with alarming statistics such as the 2% seroprevalence among dogs [7,31] and a staggering 19.5% seroprevalence among deployed American soldiers [32], underscores the pressing need for robust diagnostic measures to promptly detect and manage cases. These numbers indicate the potential threat of the disease gaining a foothold, making rapid and accurate diagnosis paramount in preventing its potential establishment and risk for local transmission.

Expanding on the diagnostics for Leishmaniasis, it is crucial to note the existing limitations and recent strides in diagnostic approaches. The scarcity of FDA-approved Nucleic Acid Amplification Tests (NAATs) for *Leishmania* in the US has created a restricted diagnostic environment with only 1 test (SMART Leish) available through the Department of Defense [33]. Limited progress in diagnostics prevails, with only a few reference labs, such as the Centers for Disease Control and Prevention (CDC), Walter Reed Army Institute, and University of Washington, providing *Leishmania* testing in North America. While a few serological-based tests have surfaced, molecular testing options remain scarce.

In a recent evaluation at Mount Sinai’s Molecular Microbiology Lab, we developed a quantitative real-time PCR (qPCR) assay targeting the 18S gene across diverse clinical samples spanning the cutaneous-visceral disease spectrum (see S1 Table) [34]. The outcomes were promising. This qPCR assay exhibited a broad dynamic range and an impressive sensitivity of 100%, detecting as few as 1 parasite equivalent per milliliter (eq-p/mL) for all *Leishmania* species [34]. Importantly, it displayed high specificity for *Leishmania* DNA, discerning it from other related microorganisms. In clinical use, this qPCR assay aligned with reference methods, accurately estimating parasite levels in both visceral and cutaneous leishmaniasis cases [34].

The attributes of this assay, including its sensitivity, specificity, and ability to gauge parasitemia, offer hope for improved leishmaniasis diagnosis and monitoring. However, further research is vital to advance *Leishmania* molecular diagnostics for broader clinical impact, including species-specific identification in order to inform treatment and management decisions. These diagnostic strides underscore the urgency of accessible and dependable testing options, especially given potential regulatory hurdles from the FDA’s proposed ruling.

## Blood parasites (*Babesia* sp. and *Plasmodium* sp.)

The increasing prevalence of Babesiosis across several US states [35] stresses the critical demand for dedicated diagnostic tests tailored to this condition. As cases surge, especially in regions such as Connecticut, Maine, Massachusetts, New Hampshire, New Jersey, New York, Rhode Island, and Vermont [35], accessible diagnostic tools become imperative.

Babesiosis manifests with varying severity, from asymptomatic cases to potentially life-threatening illness. Hence, accessible and targeted diagnostic tests are essential. The identification of *Babesia microti* in tick vectors within previously nonendemic states like Vermont, Maine, and New Hampshire [35] emphasizes the urgent need for expanded testing capabilities in these areas.

Ensuring widespread and accessible testing in endemic states is vital for swift diagnosis, timely intervention, and treatment. This highlights the necessity for developing and implementing widely available diagnostic measures specific to *Babesia* infection. Such initiatives can significantly aid in early detection, prompt treatment initiation, and better management of this increasingly prevalent tick-borne disease.

Additionally, the CDC has observed a steady rise in reported malaria cases over recent decades. The majority of these cases are imported, primarily affecting individuals traveling from countries with active malaria transmission, notably from regions in sub-Saharan Africa and South Asia [36].

While locally transmitted malaria within the USA remains relatively uncommon, the potential risk of its introduction and establishment raises public health concerns [36]. Recorded instances of autochthonous malaria transmission in the country amplify these concerns. The recent CDC alert identifying locally acquired malaria cases, particularly involving *Plasmodium vivax*, in 3 US states—Florida, Texas, and Maryland—[8] intensifies this worry.

Considering dynamic shifts in climate and environmental conditions and the presence of malaria-carrying species capable of inciting locally transmitted outbreaks across the US, heightened awareness is imperative. Equally crucial is ensuring rapid and accurate diagnosis tailored to specific malaria species, given malaria’s classification as a medical emergency. This underscores the pressing need for increased vigilance and efficient diagnostic tools to address the evolving landscape of malaria threats within the US.

Recently, our Laboratory has developed a *Babesia* and *Plasmodium* species-specific qualitative PCR, enabling the multiplex detection of clinically relevant species of these hemoparasites. This test has received provisional approval from NYSDOH (see S1 Table). Both these tests align with the necessity for sensitive, specific, rapid, robust, and comprehensive blood parasite diagnostic platforms. They also serve as pivotal tools for integrated surveys and screening, reducing the time from initial diagnosis at the patient care setting to molecular confirmation from state reference laboratories.

## Discussion

The Mount Sinai Health System encompasses 8 hospital campuses and a broader network of ambulatory facilities serving one of the most diverse populations in the world throughout the greater New York metropolitan area. Hence, from our experience, enabling academic and clinical laboratories like ours to advance assay technology while meeting regulatory requirements is pivotal. This progression strengthens the broader diagnostic community’s needs in facing emerging and reemerging neglected pathogens that can overwhelm FDA capacities. Continuing this development pipeline is crucial for effectively addressing the challenges posed by evolving pathogens.

Access to diagnostics plays a key role in combating NTDs on a global scale and addressing the recent resurgence of these afflictions within the US. Throughout history, NTDs have been a significant focus of the US’s global health initiatives. The FDA has been instrumental in ensuring access to safe and effective diagnostics. Operating within legal frameworks and regulatory functions, the FDA has implemented pathways and programs to expedite and facilitate the availability of diagnostic tools for NTDs. Notably, the United States Agency for International Development (USAID) Neglected Tropical Disease Control Program, established in response to the FY2006 Foreign Operations Appropriations Act, stands as a critical initiative in the integrated response to controlling these diseases [37].

In 2006, the introduction of the tropical disease Priority Review Voucher (PRV) program by Ridley and colleagues paved the way for significant policies [38]. The subsequent addition of Section 524 to the Federal Food, Drug, and Cosmetic Act in 2007 enhanced the FDA’s ability to grant priority review, expediting product approvals related to tropical diseases [39].

While emphasis was historically placed on treatments, the lack of diagnostic tools for NTDs gained traction as a pressing issue. Initiatives such as the Foundation for Innovative Diagnostics (FIND), established in 2003, aimed to accelerate the development and delivery of affordable diagnostic tests [38]. The 2030 NTD Roadmap also underscored the scarcity of diagnostic tools as a significant hurdle to controlling these diseases. In response, the FDA initiated the Neglected/Tropical Disease Initiative, supporting diagnosis, treatment, and prevention through rapid regulatory pathways and efficient clinical trial approaches [39].

Despite these initiatives, NTD diagnostics face challenges. The FDA’s proposed ruling on LDTs presents uncertainty about its impact on NTDs. Concerns arise about diminished financial support, leading to academic laboratories bridging gaps in NTD diagnostics using the LDT pathway. This, especially considering the development of emerging technologies and tests, is where there is a potential for advancing our understanding of NTDs, particularly in the context of the advent of next-generation sequencing (NGS) platforms. Financial constraints could impede clinical and academic laboratories’ ability to navigate the rigorous regulatory processes, hindering the advancement of NTD diagnostics.

Also, despite significant efforts by the FDA to expedite the development and review process for reliable NGS-based tests, challenges persist due to the rapidly evolving nature of these platforms. While progress has been made in providing guidance for assessing the validity of human genetic variants and germline mutations [40,41], the regulatory framework for microbial NGS-based diagnostics lags behind. Nevertheless, steps are being taken, such as the establishment of the FDA database for reference-grade microbial sequences (FDA-ARGOS) [42] and the issuance of proposed draft guidance on “*Infectious Disease Next Generation Sequencing Based Diagnostic Devices*: *Microbial Identification and Detection of Antimicrobial Resistance and Virulence Markers*” [43]. This guidance aimed to assist in the development and clinical performance of NGS-based diagnostic tests for microbial identification and detection of antimicrobial resistance and virulence markers, with significant gaps that were highlighted at that time by a multidisciplinary panel including experts from the American Society for Microbiology (ASM), Association for Molecular Pathology (AMP), Association of Public Health Laboratories (APHL), Infectious Disease Society of America (IDSA), and the Pan-American Society for Clinical Virology (PASCV) [44]. These additional regulatory layers add to the complexity of the current regulatory process for NGS-based diagnostics. This complexity may compel developers of LDTs to navigate the proposed FDA regulatory pathway, potentially resulting in delays in the adoption of NGS technologies for diagnosing tropical diseases in clinical settings. It is also noteworthy that the majority of available resources tend to prioritize bacterial and viral pathogens, often overlooking clinically relevant parasitic agents and relegating them to a secondary position, hence halting efforts like those carried out by our group devoted to support the efficient development in NGS-based parasite diagnostics [45–47].

Collaboration among laboratory stakeholders remains vital in advancing the regulatory framework for LDTs. The merger of LDTs and IVDs under FDA oversight has been debated, recognizing the distinct suitability of each assay type in varied environments. However, FDA-imposed regulatory barriers could stifle innovation and delay responses to emerging infectious diseases, amplifying test costs and complexity within the diagnostic landscape.

While we acknowledge that the FDA may not intend to regulate LDTs used solely for research purposes, we believe it is important to underscore the potential broader implications of the Proposed Rule on access to diagnostic tools for diseases disproportionately affecting marginalized populations. The absence of economic incentives for developing LDTs for NTDs affecting resource-limited and socioeconomically disadvantaged groups may result in the demise of such initiatives. Consequently, this could widen the inequality gap and limit access to testing for underserved populations, exacerbating the extrapolation of Dr. Paul Farmer’s concept of the “great epi divide” in the clinical laboratory sciences field.

By raising awareness of these issues, we aim to contribute to informed discussions and advocacy efforts aimed at ensuring equitable access to essential diagnostic technologies.

Numerous influential organizations in the infectious disease diagnostic community, such as the IDSA, the ASM, and the AMP, have raised concerns about the proposed ruling. We share their sentiment that this rule should be paused. The FDA’s move toward centralizing the diagnostic regulatory framework threatens to impede the development of NTD diagnostic tools, not only nationally but also with an effect on a global scale in contributing to the 2021–2030 NTD road map targets and by impacting the US longstanding legacy in the fight against NTDs.

The NTD road map refers to a strategic plan aimed at addressing NTDs globally within the timeframe of 2021 to 2030. This plan outlines specific targets and objectives to improve the prevention, diagnosis, treatment, and control of NTDs, particularly in low-resource settings where these diseases disproportionately affect marginalized populations. The FDA’s move toward centralizing the diagnostic regulatory framework poses a significant challenge to the development of diagnostic tools for NTDs. This centralized approach not only affects the US but also has global implications, as it may hinder progress toward achieving the targets outlined in the NTD road map. Diagnostic tools are essential for early detection, surveillance, and monitoring of NTDs, and any obstacles to their development can impede efforts to control and eliminate these diseases worldwide. Furthermore, the impact of the FDA’s regulatory changes on the US’ long-standing legacy in the fight against NTDs cannot be overstated. The US has played a significant role in supporting research, innovation, and interventions to combat NTDs both domestically and internationally. Any disruptions to this legacy, such as restrictions on the development of diagnostic tools, could undermine past achievements and future progress in addressing NTDs.

In our opinion, focus should shift toward supporting and enhancing the oversight framework for high-complexity clinical laboratory-developed testing procedures. This can be achieved through a reform of the CLIA, collaborating with other regulatory entities such as CAP, and working closely with state agencies like NYSDOH. This collaborative effort aims to harmonize clinical laboratory testing, ensuring a robust, cost-effective, streamlined, and flexible regulatory ecosystem.

The current regulatory framework, comprising CLIA regulations, CAP certification, and state DOH programs, provides a rigorous yet adaptable environment for LDTs. However, the FDA’s efforts to centralize LDT oversight pose a risk of hindering future access to timely diagnostics, particularly concerning the resurgence of NTDs in the US. The outlook is concerning, as dwindling investments from low to nonprofits may lead private sectors to withdraw funding. Academic-based laboratories, like ours, may be compelled to bridge gaps in NTD diagnostics, but navigating the Premarket Approval or 510(k) process could prove prohibitively expensive. This could ultimately impede the development of NTD diagnostic tools, negatively affecting patient care and limiting solutions for long-standing unmet diagnostic needs, especially in light of the resurgence of NTDs in the US.

## Supporting information

S1 Table List of the primary Laboratory Developed Tests (LDTs) for Neglected Tropical and Emerging Infectious Diseases developed by the Mount Sinai Molecular Microbiology Laboratory and approved by the New York State Department of Health (NYSDOH). These tests can be accessed using the project name via https://www.wadsworth.org/regulatory/clep/approved-ldt.
(DOCX)

## References

[1] Dien Bard J, Babady NE. The Successes and Challenges of SARS-CoV-2 Molecular Testing in the United States. Clin Lab Med. 2022;42(2):147–160. doi: 10.1016/j.cll.2022.02.007 35636819 PMC8901381

[2] Okeke IN, Ihekweazu C. The importance of molecular diagnostics for infectious diseases in low-resource settings. Nat Rev Microbiol. 2021;19(9):547–548. doi: 10.1038/s41579-021-00598-5 34183821 PMC8237771

[3] Hotez PJ, Jackson LS. US Gulf Coast states: The rise of neglected tropical diseases in "flyover nation". PLoS Negl Trop Dis. 2017;11(11):e0005744. doi: 10.1371/journal.pntd.0005744 29145403 PMC5689823

[4] Reardon S. What does the future look like for monkeypox? Nature. 2022;610(7931):250–252. doi: 10.1038/d41586-022-03204-7 36224412

[5] Ryff KR, Rivera A, Rodriguez DM, Santiago GA, Medina FA, Ellis EM, et al. Epidemiologic Trends of Dengue in U.S. Territories, 2010–2020. Morbidity and mortality weekly report Surveillance summaries (Washington, DC: 2002). 2023;72(4):1–12. doi: 10.15585/mmwr.ss7204a1 37192141 PMC10208330

[6] Paniz Mondolfi AE, Madigan R, Perez-Garcia L, Sordillo EM. Chagas Disease Endemism in the United States. Clin Infect Dis. 2020;70(4):717–718. doi: 10.1093/cid/ciz465 31152590

[7] Curtin JM, Aronson NE. Leishmaniasis in the United States: Emerging Issues in a Region of Low Endemicity. Microorganisms. 2021;9(3). doi: 10.3390/microorganisms9030578 33799892 PMC7998217

[8] Important Updates on Locally Acquired Malaria Cases Identified in Florida, Texas, and Maryland: CDC Health Alert Network; 2023 [updated 2023/8/28]. Available from: https://emergency.cdc.gov/han/2023/han00496.asp.

[9] Konnick EQ, Laser J, Weck KE. The Role of Clinical Laboratories in Emerging Pathogens-Insights From the COVID-19 Pandemic. JAMA Health Forum 2021;2(10):e213154. doi: 10.1001/jamahealthforum.2021.3154 36218899

[10] Emmadi R, Boonyaratanakornkit JB, Selvarangan R, Shyamala V, Zimmer BL, Williams L, et al. Molecular methods and platforms for infectious diseases testing a review of FDA-approved and cleared assays. J Mol Diagn. 2011;13(6):583–604. doi: 10.1016/j.jmoldx.2011.05.011 21871973 PMC3194051

[11] Title 21—Food and Drugs. Code of Federal Regulations. 2019.

[12] Hata J, Madej R, Babady NE. What Every Clinical Virologist Should Know About The VALID Act On Behalf of the Pan-American Society for Clinical Virology Clinical Practice Com. Journal of clinical virology: the official publication of the Pan American Society for Clinical Virology. 2021;141:104875. doi: 10.1016/j.jcv.2021.104875 34243115 PMC8513932

[13] Title 42—Part 493.17. Test Categorization. Code of Federal Regulations. U.S. Government Publishing Office; 2020.

[14] Draft Guidance for Industry, Clinical Laboratories, and FDA Staff. In Vitro Diagnostic Multivariate Index Assays. Food and Drug Administration; 2007. Available from: https://www.fda.gov/regulatory-information/search-fda-guidance-documents/in-vitro-diagnostic-multivariate-index-assays-draft-guidance-industry-clinical-laboratories-and-fda.

[15] Draft Guidance for Industry, Food and Drug Administration Staff, and Clinical Laboratories. Framework for Regulatory Oversight of Laboratory Developed Tests (LDTs) U.S. Food and Drug Administration. Food and Drug Administration. 2014.

[16] Draft Guidance for Industry, Food and Drug Administration Staff, and Clinical Laboratories. FDA Notification and Medical Device Reporting for Laboratory Developed Tests (LDTs). Food and Drug Administration; 2014. Available from: https://www.fda.gov/regulatory-information/search-fda-guidance-documents/fda-notification-and-medical-device-reporting-laboratory-developed-tests-ldts.

[17] Diagnostic Accuracy and Innovation Act Discussion Draft. US 115th Congress; 2017.

[18] S.3404- VALID Act of 2020. U.S. 116th Congress; 2020.

[19] Budelier MM, Hubbard JA. The regulatory landscape of laboratory developed tests: Past, present, and a perspective on the future. J Mass Spectrom Adv Clin Lab. 2023;28:67–69. doi: 10.1016/j.jmsacl.2023.02.008 36880010 PMC9985058

[20] Genzen JR, Mohlman JS, Lynch JL, Squires MW, Weiss RL. Laboratory-Developed Tests: A Legislative and Regulatory Review. Clin Chem. 2017;63(10):1575–1584. doi: 10.1373/clinchem.2017.275164 28687634

[21] Genzen JR. Regulation of Laboratory-Developed Tests. Am J Clin Pathol. 2019;152(2):122–131. doi: 10.1093/ajcp/aqz096 31242284 PMC6610067

[22] Emergency use authorization (EUA) Summary. Mount Sinai SARS-CoV-2 Assay (The Mount Sinai Hospital, Center For Clinical Laboratories). Food and Drug Administration; 2021 [updated 2021/8/23]. Available from: https://www.fda.gov/media/151764/download.

[23] New York State Department of Health WC. Test Approval. Available from: https://www.wadsworth.org/regulatory/clep/clinical-labs/obtain-permit/test-approval.

[24] Sharfstein JM, Becker SJ, Mello MM. Diagnostic Testing for the Novel Coronavirus. JAMA. 2020;323(15):1437. doi: 10.1001/jama.2020.3864 32150622

[25] Aden TA, Blevins P, York SW, Rager S, Balachandran D, Hutson CL, et al. Rapid Diagnostic Testing for Response to the Monkeypox Outbreak—Laboratory Response Network, United States, May 17-June 30, 2022. MMWR Morb Mortal Wkly Rep. 2022;71(28):904–907. doi: 10.15585/mmwr.mm7128e1 35834423 PMC9290387

[26] Paniz-Mondolfi A, Guerra S, Muñoz M, Luna N, Hernandez MM, Patino LH, et al. Evaluation and validation of an RT-PCR assay for specific detection of monkeypox virus (MPXV). J Med Virol. 2023;95(1):e28247. doi: 10.1002/jmv.28247 36271493

[27] Patiño LH, Guerra S, Muñoz M, Luna N, Farrugia K, van de Guchte A, et al. Phylogenetic landscape of Monkeypox Virus (MPV) during the early outbreak in New York City, 2022. Emerg Microbes Infect. 2023;12(1):e2192830. doi: 10.1080/22221751.2023.2192830 36927408 PMC10114986

[28] Miller RR, Montoya V, Gardy JL, Patrick DM, Tang P. Metagenomics for pathogen detection in public health. Genome Med. 2013;5(9):1–14. doi: 10.1186/gm485 24050114 PMC3978900

[29] Ramírez JD, Cao L, Cruz-Saavedra L, Hernandez C, Castañeda S, Muñoz M, et al. Pan-stage real-time PCR for quantitation of Trypanosoma cruzi parasitic loads in blood samples. Int J Infect Dis. 2022;122:310–312. doi: 10.1016/j.ijid.2022.06.006 35690365

[30] Franssen SU, Sanders MJ, Berriman M, Petersen CA, Cotton JA. Geographic Origin and Vertical Transmission of *Leishmania infantum* Parasites in Hunting Hounds, United States. Emerg Infect Dis. 2022;28(6). doi: 10.3201/eid2806.211746 35608628 PMC9155895

[31] Duprey ZH, Steurer FJ, Rooney JA, Kirchhoff LV, Jackson JE, Rowton ED, et al. Canine visceral leishmaniasis, United States and Canada, 2000–2003. Emerg Infect Dis. 2006;12(3):440–446. doi: 10.3201/eid1203.050811 16704782 PMC3291440

[32] Mody RM, Lakhal-Naouar I, Sherwood JE, Koles NL, Shaw D, Bigley DP, et al. Asymptomatic Visceral Leishmania infantum Infection in US Soldiers Deployed to Iraq. Clin Infect Dis. 2019;68(12):2036–2044. doi: 10.1093/cid/ciy811 30239631 PMC6769235

[33] Phillips C. FDA Approves the SMART Leish PCR assay for Diagnosing Leishmania in Individuals with Signs and Symptoms of Leishmaniasis. USAMMDA Public Affairs; 2017.

[34] Ramírez JD, Cao L, Castillo-Castañeda AC, Patino LH, Ayala MS, Cordon-Cardo C, et al. Clinical performance of a quantitative pan-genus Leishmania Real-time PCR assay for diagnosis of cutaneous and visceral leishmaniasis. Pract Lab Med. 2023;37:e00341. doi: 10.1016/j.plabm.2023.e00341 37842331 PMC10570565

[35] Swanson M, Pickrel A, Williamson J, Montgomery S. Trends in Reported Babesiosis Cases—United States, 2011–2019. MMWR Morb Mortal Wkly Rep. 2023;72(11):273–277. doi: 10.15585/mmwr.mm7211a1 36928071 PMC10027409

[36] Dye-Braumuller KC, Kanyangarara M. Malaria in the USA: How Vulnerable Are We to Future Outbreaks? Curr Trop Med Rep. 2021;8(1):43–51. doi: 10.1007/s40475-020-00224-z 33469475 PMC7808401

[37] Mukherjee S. The United States Food and Drug Administration (FDA) regulatory response to combat neglected tropical diseases (NTDs): A review. PLoS Negl Trop Dis. 2023;17(1):e0011010. doi: 10.1371/journal.pntd.0011010 36634043 PMC9836280

[38] Ridley DB, Ganapathy P, Kettler HE. US Tropical Disease Priority Review Vouchers: Lessons In Promoting Drug Development And Access. Health Aff (Project Hope). 2021;40(8):1243–1251. doi: 10.1377/hlthaff.2020.02273 34339239

[39] Neglected/Tropical Disease Initiative. Food and Drug Administration. Available from: https://www.accessdata.fda.gov/scripts/fdatrack/view/track_project.cfm?program=oc-chief-scientist&id=OCS-ORSI-Neglected-Tropical-Disease-Initiative.

[40] Use of Public Human Genetic Variant Databases to Support Clinical Validity for Genetic and Genomic-Based In Vitro Diagnostics. Food and Drug Administration; 2018. Available from: https://www.fda.gov/regulatory-information/search-fda-guidance-documents/use-public-human-genetic-variant-databases-support-clinical-validity-genetic-and-genomic-based-vitro.

[41] Considerations for Design, Development, and Analytical Validation of Next Generation Sequencing (NGS)—Based In Vitro Diagnostics (IVDs) Intended to Aid in the Diagnosis of Suspected Germline Diseases. Food and Drug Administration; 2018. Available from: https://www.fda.gov/regulatory-information/search-fda-guidance-documents/considerations-design-development-and-analytical-validation-next-generation-sequencing-ngs-based.

[42] Sichtig H, Minogue T, Yan Y, Stefan C, Hall A, Tallon L, et al. FDA-ARGOS is a database with public quality-controlled reference genomes for diagnostic use and regulatory science. Nat Commun. 2019;10(1):3313. doi: 10.1038/s41467-019-11306-6 31346170 PMC6658474

[43] Infectious Disease Next Generation Sequencing Based Diagnostic Devices: Microbial Identification and Detection; of Antimicrobial Resistance and Virulence Markers; Draft Guidance for Industry and 7 Food and Drug Administration Staff; Availability. Food and Drug Administration; 2016. Available from: https://fda.report/media/98093/infectious-disease-nextgen-sequence.pdf.

[44] IDSA. Re: Docket No. FDA-2016-D-0971 for “Infectious Disease Next Generation Sequencing Based Diagnostic Devices: Microbial Identification and Detection of Antimicrobial Resistance and Virulence Markers; Draft Guidance for Industry; Availability.” 2016 [updated 2016/9/11]. Available from: https://www.idsociety.org/globalassets/idsa/policy—advocacy/current_topics_and_issues/diagnostics/agency-efforts/091116-sign-on-letter-re-fda-guidance-on-next-generation-sequencing.pdf.

[45] Patiño LH, Castillo-Castañeda AC, Muñoz M, Jaimes JE, Luna-Niño N, Hernández C, et al. Development of an Amplicon-Based Next-Generation Sequencing Protocol to Identify Leishmania Species and Other Trypanosomatids in Leishmaniasis Endemic Areas. Microbiol Spectr. 2021;9(2). doi: 10.1128/Spectrum.00652-21 34643453 PMC8515931

[46] Patiño LH, Ballesteros N, Muñoz M, Jaimes J, Castillo-Castañeda AC, Madigan R, et al. Validation of Oxford nanopore sequencing for improved New World Leishmania species identification via analysis of 70-kDA heat shock protein. Parasit Vectors. 2023;16(1):458. doi: 10.1186/s13071-023-06073-9 38111024 PMC10726620

[47] Cruz-Saavedra L, Ospina C, Patiño LH, Villar JC, Sáenz Pérez LD, Cantillo-Barraza O, et al. Enhancing Trypanosomatid Identification and Genotyping with Oxford Nanopore Sequencing: Development and Validation of an 18S rRNA Amplicon-Based Method. J Mol Diagn. 2024. doi: 10.1016/j.jmoldx.2024.01.012 38360211

