# Supplementary material for: FDA’s proposed rule and its regulatory impact on emerging and reemerging neglected tropical diseases in the United States
Source: PLoS Negl Trop Dis. 2024 May 9;18(5):e0012116. doi: 10.1371/journal.pntd.0012116 (PMC11081280; doi:10.1371/journal.pntd.0012116)
Supplement: S1 Table — These tests can be accessed using the project name via https://www.wadsworth.org/regulatory/clep/approved-ldt. (DOCX) [file pntd.0012116.s001.docx]

**Supplementary Table 1.** List of the primary Laboratory Developed Tests (LDTs) for Neglected Tropical and Emerging Infectious Diseases developed by the Mount Sinai Molecular Microbiology Laboratory and approved by the New York State Department of Health (NYSDOH). These tests can be accessed using the project ID via <https://www.wadsworth.org/regulatory/clep/approved-ldt>

| **Facility Name** | **Facility State** | **Analyte** | **Method** | **Specimen Type** | **Permit Category** |
| --- | --- | --- | --- | --- | --- |
| Mount Sinai Laboratory | NY | Mpox virus detection | Real-time PCR | Lesions: lesion fluid on dry swab; Lesions: lesion fluid swab in VTM | Virology |
| Mount Sinai Laboratory | NY | *Leishmania* spp DNA detection | Real-time PCR | lesions: skin lesion swab; tissue: FFPE; whole blood | Parasitology |
| Mount Sinai Laboratory | NY | *Trypanosoma cruzi* detection | Real-time PCR | Whole blood | Parasitology |
| Mount Sinai Laboratory | NY | *Plasmodium* DNA detection | Real-time PCR | Whole blood | Parasitology |
| Mount Sinai Laboratory | NY | *Babesia* DNA detection | Real-time PCR | Whole blood | Parasitology |
